# Winchester County Hospital
*Provincial Medical Gazette, No. I.


**Published:** 1829-07-01

**Authors:** 


### CLINICAL REVIEW.

### IX.

### WINCHESTER COUNTY HOSPITAL.*

[Mr. George Miller, hospital pupil, Reporter.]

Nothing can give us more unfeigned satisfaction than witnessing the publication of the many interesting cases which occur, as occur they constantly must, in our county hospitals and provincial infirmaries. Those who best know the medical officers attached to these institutions, also know that in point of information and talents, they are nothing behind their metropolitan confrères, though hitherto, we must say that they have slumbered at their posts, and not displayed the zeal they should, in publicly promoting the cause of our noble science, and contributing their share to "that store of facts, by which disputed points of doctrine may be decided, wavering opinions confirmed, and mistaken theories refuted." We trust that a happier æra is approaching, nay, is even now begun, when disgraceful and gladiatorial conflicts amongst medical men, shall give way to an ardent co-operation in improving the profession, instead of debasing it. Dropping, however, these preliminary remarks, we come to notice some cases reported in a very creditable manner indeed, by Mr. George Miller, in our newly-born provincial contemporary. It would seem by the table of medical

* Provincial Medical Gazette, No. I.

and surgical patients received in the course of a quarter in the Winchester hospital, that the pupils must have very considerable opportunities of studying the practical part of their profession within its walls. From what we have seen of young men who have come to this town to complete their education, we should say, that the county hospitals were very excellent schools indeed.

With regard to the present report, we shall follow our usual plan of selecting particular cases, and freely appending what remarks occur to our minds upon each.

### I. PARALYSIS OF THE UPPER EXTREMITIES.

A man, ætatis 67, was mowing in a field when suddenly he was seized with such a loss of power in his arms as compelled him to desist. He applied no remedy, but the feebleness continued, and five weeks after this attack, he entered the hospital. His health at this time was good, he had no cephalalgia, but like an intoxicated person he could not maintain the erect posture. The deltoid muscle of both sides, and especially the right, were quite wasted away, and the shoulder sunk as in dislocation.

"The depleting system, as cupping, blistering, &c., was first resorted to, without the least benefit, and this was altered to the diffusible stimulants; but the patient being desirous to return home, his friends removed him in a cart; and he had not left the hospital long, before he became so much worse that they judged it expedient to return with him. When he arrived, he was in a complete state of collapse, extremities cold, pulse scarcely perceptible, lips livid, and his breathing extremely laborious, but he was sensible. He rallied on the proper application of remedies; and became much better towards evening, but during the night he suffered a relapse, and expired the next morning."

On dissection, the vessels of the brain were found turgid with blood, and there was a varicose enlargement of the veins on the outer side of the plexus choroides. The frontal sinus was filled with unusually thick pus, but no other appearance of disease was detected in the body, though the spinal marrow does not appear to have been examined.

It is pretty clear, we think, that nothing exists on the face of the dissection to account satisfactorily either for the paraplegia, or the final and fatal result. The ignorance, however, in which we are left respecting the state of the spinal marrow, puts a stop at once to any speculations on the proximate cause of the disease.

### II. AMPUTATION FOR DISEASE OF THE KNEE-JOINT — DEATH — ABSCESS IN THE LUNG.

William Triggs, ætatis 49, a gamekeeper, was admitted on the 24th of September, with disease of the right knee-joint, of three years' standing. He had had a severe attack of fever, after which he experienced a considerable degree of weakness in the joint, for the space of twelve months, when, without any obvious exciting cause, it began to enlarge and grew extremely painful. He continued, however, his usual avocations until about two months prior to his admission, when the disease was much exasperated by receiving a heavy blow on the inner condyle of the femur, which immediately occasioned a severe attack of inflammation of the knee, and swelling extending down the leg and foot. His general health had not suffered much, nor had he applied for advice, till a month or so prior to admission.

"On examination, the natural appearance of the joint was completely lost in the general tumefaction, the temperature of the part much increased, unaccompanied with redness. The slightest pressure occasions great deep-seated pain in the joint, reaching down to the foot. There is no motion at the knee, and the whole extremity appears cumbersome and heavy. The thigh above the knee is diminished in size, but the leg and foot enlarged. His general health is not so much affected as would be anticipated from the nature and extent of the disease. Pulse 60; bowels regular; countenance natural, though the tongue is whitish and furred at the back part, and there is a degree of emaciation indicative of constitutional irritation. During the night the pain and increase of heat become greatly aggravated, which, with the violent twitchings of the limb, almost preclude the possibility of sleep."

We need not detail the items of the treatment employed, suffice it to say, that it had no effect in arresting the disease; on the contrary, the patient's symptoms, general and local, were aggravated so much on the 7th October, that it was thought proper to remove the limb, to which he readily assented. Amputation was accordingly performed above the knee, in the usual manner, seven arterial branches requiring to be tied. The patient lost very little blood, and suffered extremely little. He was removed to bed, and took 30 minims of laudanum in a glass of camphor mixture.

On examining the amputated limb, there was found on the posterior part of the joint, behind the capsular ligament, but entirely external to it, an abscess containing about six drachms of healthy pus. The "synovial gland" (qy. what is that?) was entirely destroyed, the cartilages more or less absorbed and disorganized, the bones, and especially the rotula, highly inflamed, the cellular substance of the leg and foot filled with serous fluid. From the post mortem appearances it is evident, as the history of the case would à priori lead one to infer, that the disease was essentially ulceration of the cartilages, and the inflammation of the bones a secondary lesion.

He went on tolerably well for the first three or four days, the pulse ranging from 90 to 100, the temperature of the skin being little increased, the bowels being freely opened by aperient powders, and only a little nausea being experienced, which was checked by the effervescing draught with a few drops of laudanum. The discharge from the stump, which exuded through the dressings in considerable quantity, was so fetid as to require the application of rags moistened with the chloride of lime. On the 11th, four days from the operation, the dressings were removed for the first time, when the edges of the wound were found to be adherent, except at its extremities, through which much grumous blood had escaped. In the evening of the 12th he was attacked with violent retchings, passed a restless night, and next day presented these symptoms; constant and violent retching, distention and pain of the abdomen increased on the slightest pressure, quick and feeble pulse, clean tongue, extremely anxious countenance, and great exhaustion. On dressing the stump again, the adhesions were found to be completely broken up, and a still greater quantity of grumous blood was discharged. Warm brandy and water was given immediately, and frequently repeated, and a draught of opium, carbonate of ammonia, tincture of bark, and camphor mixture, was ordered to be taken every four hours. The patient, however, did not rally, and although the stump itself assumed a very healthy appearance, he sank on the 26th.

"On dissection, the only sign of disease, either in thoracic or abdominal viscera, was a *small abscess* in the right lung, all the rest exhibiting the most perfect health."

We cannot but express our regret that the nature, that is, the anatomical features of the abscess in the lung, was not more minutely and definitely described. From the statement in our contemporary, no one could positively determine, whether the abscess was a common vomica from suppurating tubercles, a genuine phlegmonous abscess in the lung, or one of those purulent depôts, of which we hear and see so much, and know so very very little. For our own parts, however, we are strongly inclined to believe that the "abscess" was one of the latter description, and this, not merely because there existed matter in the lung, but rather from the nature of the symptoms during life. The violent retchings, the quick and weak pulse, the anxious countenance, and extreme prostration of the nervous system, occurring a certain number of days after the performance of a capital operation, are very characteristic features of the formation of pus in the parenchyma of the lungs or liver.

In these cases, however, there are always rigors, as far as we have seen, which are not alluded to in the present instance, and the tongue, instead of continuing clean, becomes dry, with a deep brown fur in its centre and a morbidly red appearance at its sides and tip. On the whole, however, as we said before, we are strongly inclined to believe that the case was one of those purulent deposits, and again express our regret, that the notes of the dissection should not have been given with greater minuteness. One word more before leaving the subject; we would earnestly intreat our provincial surgeons to attend to this class of affections, to look for them carefully and closely, and when they shall have found them, as find them, and that too often, we know that they will, to bring them before their professional brethren. The surgeons of the Glasgow Infirmary are deserving of much commendation for the zeal and activity they have shewn of late in cultivating this branch of surgical pathology.

III. SPONTANEOUS CURE OF FEMORAL ANEURISM—ASSISTED BY PRESSURE.

A labourer, 43 years of age, had the right femoral artery tied with success by Mr. Lyford, at the hospital, for popliteal aneurism. In June last, nearly two years after the operation, he was occupied in mowing, when suddenly he felt something give way in the thigh, accompanied with violent pain, and succeeded by a throbbing at the part, which became so much aggravated at night as to rob him of his sleep. In the course of a very short time he observed a small tumour at the lower and internal part of the thigh, which gradually increased, till he made application at the hospital, at which time it was four or five inches in circumference, circumscribed, rather hard, but entirely reduced by pressure on the artery above. He could not at this time enter the house, and was directed to apply a flannel roller so as to occasion moderate pressure, and keep himself perfectly quiet at home. On the 27th of September, he was made an in-patient, when the tumour was found to have greatly subsided, and had lost all pulsation. It now appeared that the pressure of the bandage having caused a most decided diminution of pain, he had increased the compression from time to time by tying a handkerchief tight around the thigh, the knot being placed directly over the centre of the aneurism. Three days previous to his admission, the pulsation of the tumour had totally ceased, and since that time he had experienced sensations in the limb, precisely similar to those which he had felt after the operation on the opposite. He also complained most severely of a burning heat immediately under the skin, as if from boiling water trickling down the foot and leg. The temperature of both feet was alike.

Under these circumstances, pressure was re-applied by Mr. Lyford, by means of a tourniquet and splint, and in the course of ten days, on removing the apparatus, all appearance of swelling had entirely vanished, and so had every vestige of the disease. The knee-joint was at this time capable of perfect flexion and extension, and the patient being able to walk without any support was discharged cured.

Not the least curious part of this interesting case is the rapidity with which the *debris*, if so improper a term may be permitted, of the aneurism disappeared. Even where the common operation is successfully performed, it is mostly a considerable time before the consolidated tumour is dispersed. In this case, however, it would seem from the *almost entire* subsidence of the tumour on pressing the artery above, that little coagulum existed in the sac, which would make, of course, a material difference in the length of time required for the completion of the absorptive process. The *mode* in which the spontaneous cure, if spontaneous we can strictly call it, took place, in the absence of dissection is obviously a matter of uncertainty, but still if we do not know what it *was*, we can tell what it probably was *not*. It was clearly not produced by sloughing of the sac, nor by the gradual deposition of lamellated coagulum filling up its cavity and that of the artery from which it sprung; nor yet, as occasionally happens, by the filling up with coagulum of the sac alone. Is it probable that the sac, which was rather large, became itself the immediate mean of cure, being pressed by the bandage on the artery above, so as to obstruct the circulation through it? This position or overlapping of the sac, it is well known has more than once effected the spontaneous cure of the disease, and we see no reason why it should not but several why it should have done so in the present instance.

The cases of Guattani prove that compresssion will succeed, in many cases of aneurism, and we cannot but think, that surgeons might employ it oftener than they do. When the tumour is recent, seated in the course of an artery, like the femoral, not very large, emptied, or nearly emptied by pressure, and free from inflammation, the case, in our humble opinion, is a fair one for the trial of compression, especially if the patient be in the upper ranks of life, able and willing to submit to perfect repose, both of body and of mind. By compression, we do not mean sudden or violent measures, for the pain they produce is such as no human being can endure and ten times worse than any operation, but gradual and easy pressure of the tumour and the limb, with low living and repose. It is only in particular cases that such a proceeding is either required or desired, but we think that in some, it would answer, and at all events, would prove no bar to an after operation, if it failed.

IV. Extensive Wound of the Heart—Survival of the Patient for an Hour and a Quarter.

A bricklayer's labourer, twenty-five years of age, fell from a ladder on the top of a house down upon some wooden railings, which pierced the upper part of the abdomen, and produced a wound about two inches and a half in extent, through which a vast quantity of the small intestines protruded. A surgeon, who was sent for, reduced the bowels, applied three sutures to the wound, and directed the patient to be taken to the hospital. On his admission he was found in a very exhausted state, having all the symptoms produced by a violent injury, or great internal hæmorrhage, and although he was wrapped in warm blankets, and had hot brandy and water every ten minutes, he died in rather more than one hour and a quarter after the occurrence of the fall.

On examination of the body, which took place six hours after death, the wound was seen to have extended through the diaphragm, immediately beneath the sternum, into both ventricles of the heart, and through the septum ventriculorum, leaving a large ragged opening, an inch in length, in their parietes. Through this laceration the blood had escaped into the chest, which contained rather more than two quarts of that fluid perfectly uncoagulated. The small intestines in many places were in a complete state of intus-susception, but otherwise the body was healthy.

"This case, in one point of view, is highly interesting, and of much practical importance, inasmuch as it clearly manifests, that a very considerable injury of the heart may be sustained without causing that immediate dissolution which is commonly supposed uniformly to arise from wounds of this most important and vital organ. We have on record numerous instances of rupture of this viscus from disease, occurring principally, if not entirely, amongst persons at an advanced period of life. In ten patients, who lost their lives from this organic lesion, it was observed that eight died instantly; one at the expiration of about two hours; and another at the end of fourteen. The longest period which a person has been known to survive after a wound of the heart is forty-eight hours, exemplified in the case of a soldier, on duty at Haslar hospital, who had a bayonet plunged into the right auricle. On examination after death, it was found that a coagulum had formed, and completely filled up the wound! but in an effort to evacuate the bowels, a second hæmorrhage took place, and he was found dead, sitting on the night chair."

A case of calculus in the bladder is detailed which occurred in the person of a short fat healthy looking man, and for which the operation of lithotomy was performed, and it would seem very well performed too, by Mr. W. Wickham. The gorget was introduced and the bladder incised in the space of one minute, and the stone, which weighed thirteen drachms and a half, extracted at the end of three. In a child which we saw operated on by Mr. Keate at St. George's Hospital, the whole, almost incredible as it may appear, was completed in *forty seconds*; and in another case Mr. Brodie performed the operation in something more than a minute. We know that in children lithotomy is quite a different operation from lithotomy in adults; and, further, we do not attach much importance, at the best, to surgery by the stop-watch. That operation is best done which is done with coolness, decision, and skill. When the citò can be combined with the tutè, so much the better for patient and surgeon; but, when it is not, Heaven help them both.

### V. FOOD IN THE PHARYNX FOR A YEAR AND A HALF.

A young woman, after partaking of some boiled beef at a hasty meal, was suddenly seized with excessive difficulty of deglutition and dyspnœa with cough, but without expectoration. Next morning, in consequence of the horrible sense of suffocation with which she was constantly threatened, she applied to a medical gentleman who examined the throat, but could find no extraneous substance within it, and prescribed an emetic or two, without producing any relief. From this time up to her admission into hospital, a period of a year and a half, she was forced to live entirely on fluids, by which her strength was materially impaired, and her frame emaciated greatly. After her admission, the finger was passed to the posterior part of the throat (the tongue being at the same time much depressed) into the pharynx, when a foreign body was felt at the right and inferior portion of that bag, resembling a morbid growth of the part. After some little time it was dislodged, and fell into the upper portion of the œsophagus, from which it was pushed into the stomach by the œsophagus-bougie, when immediately the power of easy deglutition returned.

Some other cases are yet to be noticed, and to these we shall return at a future opportunity.

### LIII.

### WINCHESTER COUNTY HOSPITAL.

#### I. Dissection of a Case of Ascites.*

We resume with much pleasure the review of the Report from the above hospital, in our esteemed Provincial contemporary. We have now so often expressed our sentiments on the value of *authenticated* hospital reports, that anything further on the subject would be a matter of supererogation. If, however, authentic reports are of use, what can we say of those which are *not* authentic, but that they are fraught with mischief and delusion. Medical and surgical science after all are only a superstructure bottomed upon *cases*, and if they are false, if the ground-work be rotten, then woe to the edifice reared above. It is indeed a house built upon the sand. It was with this impression and an honest regard for the welfare of medicine, that we struggled so long and so successfully against the system of reporting in a journal that is now sinking fast into oblivion. To assert that we discountenanced accurate reports, was mendacity of as deep and as dark a dye as ever polluted the pages of even that most iniquitous publication; but that we did discountenance those in the Lancet is as true as they were false. We are proud for the honour of the profession to say now, that this wholesale manufacturing of lies is looked on with no feelings but those of disgust, and that no one who sets a value on his own character would at present dream of quoting, either for his opinions or against them, the pages of the Lancet. Magna est veritas et prævaluit.

The following dissection will shew the description of appearances not unfrequently presented in the bodies of patients dying of ascites. It is no great wonder with such diseases that medicine and surgery should both be foiled. The patient, a female, had been successfully treated two years previously for dropsy, which, however, at the end of that time returned, and occasioned her re-admission into the hospital. She was getting much better when diarrhœa and hæmatemesis supervened, and carried her off in a few days.

"*Sectio Cadaveris.* On laying open the abdomen, three quarts of a yellowish, turbid, and very offensive fluid escaped, in which large portions of coa-

* Provin. Med. Gaz. No. I.

gulable lymph and pus were floating and intermixed. So extensively diseased was the peritoneal covering of the intestines, that at first view it was almost impossible to recognize the intestines themselves. The omentum and the intestines were almost inseparably united, as well as the surface of the several convolutions of the intestines with each other. The viscera and peritoneum lining the abdominal muscles were studded with tubercles and ulcerations, each covered with a black slimy pigment. On tearing through the adhesions by which the viscera were agglutinated together, large ulcerated openings were detected, communicating with the cavity of the intestines, and through which portions of fæcal matter had escaped. Mesentery healthy. Liver enlarged, and of a peculiar pallid hue; a section of this viscus exposed the ducts loaded with yellow bile. The gall bladder distended with green bile. Pancreas somewhat enlarged and indurated. Spleen natural. The peritoneum covering the stomach healthy; its pyloric extremity somewhat contracted and indurated, and its internal surface marked by red patches, apparently the abraded termination of vessels, and from which the blood which had been rejected from the stomach was effused. The pericardium contained twelve ozs. of a perfectly clear fluid. The substance of the lungs healthy, but adherent to the pleura costalis. This case in its commencement seems to have originated in a chronic inflammation of the peritoneum, which, from its having been allowed to continue, had produced that disorganization and effusion which were detected on laying open the abdomen. It is more than probable, that at the period when she first left the hospital entirely relieved from her hydropic symptoms, a slow but destructive inflammation of the peritoneum still continued its ravages, and produced both the second effusion with that extensive mischief which was the immediate cause of the patient's dissolution."

We not unfrequently, or at any rate we sometimes find aneurismal sacs proceeding from the thoracic or abdominal aorta, very nearly filled with lamellated coagulum, and therefore it would seem in progress to a natural cure. In a patient, aged 44, who died at the Winchester Hospital, an aneurismal sac was found to proceed from the arch of the aorta, and pass into the right auricle so as nearly to obliterate that cavity. The aorta itself was much dilated, and the sac was nearly filled with layers of the fibrinous part of the blood. The patient had entered the hospital merely for dyspnœa unaccompanied by pain, but suddenly typhoid symptoms came on, and in this state he died. Besides, the aneurism mentioned above, the roots of the lungs were found to be "in a diseased state." The mischief of it is that in these cases when the cure would appear to be accomplishing, the patient commonly dies from the mere irritation produced by the pressure of the solid tumour on important organs. It is not the aneurism itself which kills, at least in the thoracic portion of the aorta, so much as the inflammation and irritation induced in the bronchi and lungs. In the present case one would really be tempted to imagine that the great diminution of the calibre of the right auricle, must have had some share in producing the patient's death, and perhaps the disease in the root of the lungs.

### II. EXTENSIVE DISEASE OF THE KIDNEYS AND BLADDER.

A stout, short man, aged 52, suspected of and sounded for stone, was admitted under Mr. H. Lyford, with the following symptoms. Frequent desire to make water, with dull aching sensation at the extremity of the penis after it—urine stopping suddenly when passing in a full stream attended with the most excruciating pain and tenesmus—slight mucous discharge from the urethra—sense of weight and uneasiness in the lumbar region, evident tension in the hypogastric—bowels constipated--pulse 95—appetite good. A pint and a half of dark coloured urine was drawn off by the catheter, but the man complained that his bladder was not yet completely empty, and on passing the finger up the rectum the prostate was found twice its natural size. *Six leeches to*

*the perineum—tepid hip bath—occasional doses of castor oil. Infus. folior Buchu,* ℥x. *Tinct. Hyosciam.* ℥ss. *Sod. Carb.* ℈j. ℳ *ter die.* Complete retention of urine with occasional stillicidium came on and required the catheter twice a day. About two quarts of urine were drawn off each time, and deposited on cooling three or four ounces of pus. After the urine had been abstracted, the bladder was now injected with three grains of extract of poppies in twenty of water, which afforded a great deal of relief. On the morning of the 15th, he was seized with violent sickness and retching, which in great measure subsided under the use of an opium pill, and purging by solution of salts. On the 16th, the pulse was 100, and small; tongue dry and brown; skin hot; some nausea. An effervescing saline was administered, the bladder injected as before, and the tepid hip-bath had recourse to; but on the 18th, the countenance was quite hippocratic, a violent rigor succeeded on the 20th, with distressing sickness and great exhaustion, and he rapidly sank and died.

"*Post Mortem Examination.*—The abdomen opened in the usual way. The right kidney not enlarged, but embedded in an extraordinary quantity of adipose substance, preternaturally vascular; and its substance studded throughout with extensive purulent patches. The pelvis and ureter likewise much enlarged. The left kidney contained a large abscess filled with grumous blood and pus, and its whole structure more or less disorganised. The infundibulum and ureter both inflamed and ulcerated. The bladder was found to contain about a pint of urine, mixed with large purulent flakes and coagulated blood, much thickened; muscular fibres particularly coarse and strong; the internal coat in entire state of ulceration. It was formed into numerous sacculi, one of which, in particular, at the posterior and mesial part of the viscus, was as capacious as the bladder of a child; the opening into it circular, and about one inch and a half in circumference. It was full of the most offensive and dark coloured urine. The prostate gland enlarged, as ascertained by examination before death, twice its natural size; and evidently containing a fluid. A section of it exposed its interior converted into a semifluid pulp. Its ducts were enormously distended, and rendered completely impervious by small calculi, the size of a pea. Thoracic viscera healthy. Liver smaller than natural. Gall bladder filled with greenish bile. The duodenum appeared to have been recently inflamed. Stomach healthy."

### III. SUDDEN PROTRUSION OF THE INTESTINES INTO THE SCROTUM.

John Marsh, æt. 50, a labourer, was brought into the hospital, having just been run over by a cart laden with bricks.

"His scrotum, on inspection, was found to be of most enormous size, extending two-thirds downwards between the thighs, and measuring in circumference 17 inches. Its colour of a jet black; and its texture, from over distention, so exquisitely thin, as to threaten immediate rupture from the slightest manipulation. The abdomen perfectly flaccid and nearly empty. Immediately over the umbilicus existed a large transverse ecchymosis, indicating the exact course of the wheel over the belly. The patient was incessantly vomiting, accompanied by the most urgent retching, extremities cold, the body bedewed with profuse clammy perspirations, attended with syncope. On being placed in bed the viscera were returned to their natural situation without much difficulty, merely by elevating the hips, depressing the shoulders, and applying moderate and careful pressure with flannels moistened in hot poppy fomentation; the facility of reduction depending on the large opening through which the viscera had passed, together with favourable and relaxed state of the patient. One grain of opium was now exhibited, with a view of allaying the irritability of the stomach, and the abdomen fomented, as well as the scrotum, with poppy fomentation; bottles of hot water applied to the feet."

At 4, p. m., the sickness continued unabated, the abdomen was extremely tender, the extremities were warm, but the pulse was feeble. The opium pill was repeated; a tepid bath used for ten minutes and followed in an hour by a glyster; and the scrotum constantly supported. He passed an easy night, and next day the sickness had subsided and the tenderness was less. The glyster had operated twice; pulse 90, and feeble; skin cool; countenance not so anxious. Castor oil and the warm bath were the means employed, but he passed a bad night, and next day had intense pain in the belly upon pressure; some tension; nausea; pulse 100, and wiry; skin dry; tongue white. The scrotum was reduced in size, but completely black. Thirty leeches were immediately applied, together with the tepid bath, a blister at bed-time, and a mixture of sulphate of magnesia. Stools were obtained by the employment of these remedies, and all the foregoing formidable symptoms, quickly passed away. On the twelfth day from the occurrence of the injury the patient was reported as quite convalescent, and able to sit up for some hours in bed, the precaution of applying a double truss having previously been taken. At the end of the third week he was discharged cured.

"Since this patient has left the hospital, I am informed he is subject to occasional diarrhœa, which is extremely violent, and reduces him very considerably before it can be checked. He is compelled to wear his double truss both night and day, otherwise the viscera descend immediately into the scrotum in very large quantities. He was never afflicted with hernia prior to this unfortunate occurrence."

Here we see a patient whose viscera are forced with immense violence into the scrotum, although no hernia had existed before, recover under no more active treatment than thirty leeches and a blister. Another man will be destroyed by peritoneal inflammation merely in consequence of two or three inches of intestine having lain in a hernial sac in the scrotum for a few hours, and that though the stricture has never been severe, and the gut has been returned with tolerable ease. This is no imaginary case, for we lately witnessed it at St. George's Hospital. The patient was admitted in the morning with symptoms of strangulated scrotal hernia, which had descended in the night. Under the use of the warm bath and bleeding the intestine was easily returned by the house-surgeon, but the man was dead before nine at night. On dissection no stricture was found at the neck of the sac, nor at the ring, no rupture of gut, and no very violent peritoneal inflammation. In the present case it is impossible to conceive that the immense mass of intestine protruded into the scrotum could have received a covering or sac from the peritoneum. The latter must have been ruptured on either side, and probably the muscles gave way also, forming the *direct* hernia. The circumstance of the peritoneum being broken, whilst it increased perhaps the danger of inflammation, lessened or entirely did away with that of stricture on the viscera descending through it. The laceration on either side must have been so extensive as to make it a difficult matter to retain the intestines in the belly by such a contrivance as a double truss. We should conceive that some sort of elastic belt, which should brace up the whole hypogastrium, and still make especial pressure at the openings above or about the rings, would be much more likely to answer the end, than the instrument employed.

This closes the report from the Winchester Hospital, but we trust we shall frequently meet again in print with the able and industrious reporter.

